# Supplementary material for: Changes in Management Lead to Improvement and Healing of Equine Squamous Gastric Disease
Source: Animals (Basel). 2023 Apr 28;13(9):1498. doi: 10.3390/ani13091498 (PMC10177505; doi:10.3390/ani13091498)
Supplement: Supplementary file 1 [file animals-13-01498-s001.zip › animals-2327316-supplementary.pdf]

animals

MDPI

Supplementary table 1. Macronutrient composition (as-fed basis) of the components of the experimental ration; the compound complementary feed without collagen, hay (88.7% DM) and the mineral feed supplement

Item (as fed)	Compound complementary feed[1]	Hay[2]	Mineral feed[1]
Crude Protein (g/kg)	115	73	102
Digestible Crude Protein (VREp)[3] (g/kg)	82	53	71
Crude Fat (g/kg)	32	12	32
Crude Fiber (g/kg)	114	274	191
Crude Ash (g/kg)	77	105	108
Starch (g/kg)	220	-	95
Sugar (g/kg)	76	75	63
NDF (g/kg)	336	525	-
ADF (g/kg)	-	298	-
ADL (g/kg)	-	32	-

[1] The compound complementary feed (commercially available as "Voermeesters Basis" and mineral feed (commercially available as "Voermeesters Mineralenbikkels") was provided by Voermeesters B.V., Marsdijk 31, 4033 CC, Lienden, The Netherlands. Chemical composition of the feed without collagen as declared by the manufacturer is presented. The hydrolyzed collagen was pelleted into the compound complementary feed.

[2] Hay analysis (NIRS) performed by Eurofins Agro, Wageningen, The Netherlands (Order no. 350357/005183689)

[3] Digestible Crude Protein (VREp) values according to the Dutch Feed Evaluation system for horses [Centraal Veevoederbureau. Het EWpa en VREp systeem. CVB documentatierapport No. 31, Centraal Veevoederbureau, Lelystad, the Netherlands 497 (in Dutch), 2004.].
